# PET/CT in the evaluation of pulmonary solitary nodule

**DOI:** 10.1590/0100-3984.2016.49.2e4

**Published:** 2016

**Authors:** Felipe de Galiza Barbosa

**Affiliations:** 1 MD, Radiologist, Subspecialization in Abdominal Imaging and Hybrid Imaging Methods (PET/CT and PET/MR) at Hospital Sírio-Libanês and at Universidade de São Paulo (USP), São Paulo, SP, Brazil. E-mail: felipegaliza@gmail.com.

In recent decades, we have been faced with an increasing number of thoracic computed tomography (CT) examinations worldwide, an increase that can be partially attributed to the growing number of lung cancer screening programs. In addition, the development of scanners with higher spatial resolution has increased the number of solitary pulmonary nodules (SPNs) diagnosed per day^(1)^. Probabilistic models for predicting cancer in SPNs can be very important to facilitating patient management and avoiding unnecessary expenses. Studies have shown that ^18^F-fluorodeoxyglucose positron-emission tomography/computed tomography (FDG-PET/CT) is another valuable imaging modality for the assessment of indeterminate SPNs^(2)^, improving accuracy in the differentiation between benign and malignant nodules, as well as informing the decision-making process related to patient management.

In the previous issue of **Radiologia Brasileira**, Mosmann et al. published the article "Solitary pulmonary nodule and ^18^F-FDG PET/CT. Part 1: epidemiology, morphological evaluation and probability of cancer"^(3)^, which provides a very interesting detailed review of SPN evaluation and FDG-PET/CT. The authors discuss the background of the two components of this hybrid method (PET and CT) individually. This first part of the article presents a brief, concise overview regarding the morphologic assessment of SPNs by CT and puts all of that information into clinical perspective by presenting the current models to predict malignancy in pulmonary nodules. Other studies in the literature have discussed the morphological characteristics of SPNs and their unique cancer potential^(1,4)^. Truong et al.^(4)^ summarized the CT aspects of SPNs by subtype (solid or part-solid), correlating each with its own additional tumor risk.

In clinical practice, the management of pulmonary nodules can involve a wide variety of choices, from the imaging method to be employed to the invasive approach used in their diagnosis and treatment. The implementation of probabilistic models can be helpful in stratifying patients by cancer risk, consequently playing an important role in the clinical decision-making process. The Mosmann et al. article provides a comprehensive explanation of the models currently in clinical use^(3)^. Such an approach can have additional value while there is increasing discussion regarding the funding of health care systems in Western countries, including Brazil.

In addition to evaluating the morphology of SPNs, in recent decades there has been increasing interest in studying other parameters, such as nodule metabolism (with FDG-PET) and perfusion (with contrast-enhanced imaging studies), to predict malignancy. In this "Part 1" article^(3)^, Mosmann et al. conclude by summing up all of the important clinical information about the most widely used hybrid imaging method for metabolic evaluation, PET/ CT. The literature corroborates the important role that PET/CT plays in the evaluation of SPNs^(4-7)^, showing it to be superior to contrast-enhanced imaging methods. There is a very real possibility that PET/CT will become even more relevant with the upcoming advances in PET digital detector technology, which will provide higher spatial resolution and improve image quality^(8)^, making the PET evaluation of smaller pulmonary nodules more reliable, with acceptable accuracy.

Finally, I would like to congratulate the authors for conducting a comprehensive and practical review of such a relevant topic, especially for including probabilistic models to predict cancer in SPNs. In the context of the current discussion of health care system funding worldwide, realistic approaches to be discussed are always welcome.
